# Phenylboronic acid conjugated multifunctional nanogels with ^131^I-labeling for targeted SPECT imaging and radiotherapy of breast adenocarcinoma

**DOI:** 10.3389/fbioe.2022.973141

**Published:** 2022-07-22

**Authors:** Lingdan Kong, Jingyi Zhu, Hongxing Su, Lingzhou Zhao, Yi Lu, Meilin Zhu, Wenjie Sun

**Affiliations:** ^1^ School of Ophthalmology and Optometry, School of Biomedical Engineering, Wenzhou Medical University, Wenzhou, China; ^2^ School of Pharmaceutical Sciences, Nanjing Tech University, Nanjing, China; ^3^ Department of Nuclear Medicine, Shanghai General Hospital, Shanghai Jiao Tong University School of Medicine, Shanghai, China; ^4^ Institute of Biotechnology, RWTH Aachen University, Aachen, Germany; ^5^ School of Basic Medical Sciences, Ningxia Medical University, Yinchuan, China

**Keywords:** phenylboronic acid, nanogel, SPECT imaging, radiotherapy, breast adenocarcinoma

## Abstract

We report a new ^131^I-labeling functional platform for targeted single-photon emission computed tomography (SPECT) imaging and radiotherapy of breast adenocarcinoma. In this study, polyethyleneimine (PEI) based nanogels (P.NH_2_ NGs) were prepared by water/oil polymerization, modified with targeted agent phenylboronic acid (PBA), and labeled with radionuclide ^131^I. The NGs without ^131^I-labeling own a spherical structure, uniform size distribution, and good cell viability. After ^131^I-labeling, the obtained ^131^I-PBA-PHP NGs displayed much higher cellular uptake than the non-targeted NGs due to the good softness and fluidity of NGs and the PBA targeting. The *in vivo* results demonstrated that ^131^I-PBA-PHP NGs could specifically target breast cancer cells and efficiently aggregate into xenograft breast adenocarcinoma for tumor SPECT imaging and specific radiotherapy. The developed ^131^I-labeling NGs may be used as a promising platform for efficient radioactive theranostic nanoplatform of tumor.

## Introduction

The rapid development of nanotechnology and the in-depth understanding of tumor biology have promoted the application of nanotechnology in the field of tumor molecular medicine, especially in the fields of medical imaging (Li et al., 2017; Qi et al., 2018; Siddique and Chow, 2020), tumor molecular diagnosis (Combes et al., 2021; Wang et al., 2022) and anticancer therapy (Qin et al., 2017; Cheng et al., 2021; Li et al., 2022a). In recent years, based on the continuous development of nanomaterials, the organic combination of diagnosis and treatment can be achieved through the design of a variety of nano-drug systems, so as to realize the integrated treatment of tumor diagnosis and treatment (Zhu et al., 2021). Nuclear medicine can realize real-time imaging and specific treatment of tumor simultaneously with radionuclides and radio-labeled compounds. Single-photon emission computed tomography (SPECT) is one of the most important methods in nuclear medical imaging, which has the characteristics of high sensitivity, functional imaging and quantitative diagnosis (Fu et al., 2020). Among the radionuclides commonly used in clinical practice, iodine-131 (^131^I) has the advantages of easy labeling, long half-life (t_1/2_ = 8.01 d) and suitable radiation ability compared with other radionuclides, which can be used in SPECT imaging and radiotherapy simultaneously.

However, radionuclides have shortcomings such as short blood circulation time and poor specificity *in vivo*. Some studies indicate that the appropriate nanocarrier system of radionuclides can be an effective vehicle to achieve enhanced tumor accumulation (Li et al., 2016). Therefore, the combination of radionuclide and nanocarrier enables radionuclides efficiently to distribute in target tissues, and further achieve accurate imaging and effective treatment in target tissues, especially in the terms of tumor theranostic (Pellico et al., 2021). There is a wide variety of nanomaterials, and nanomaterials used in nuclear medicine are constantly updated, including dendritic macromolecules (Ghoreishi et al., 2017; Li et al., 2019), polymer micelles (Ramos Oda et al., 2017; Aranda-Lara et al., 2021), microspheres (Wu et al., 2022), and nanogels (NGs) (Drude et al., 2017; Li et al., 2021a; Li et al., 2022b). Among them, NGs are hydrogel particles with three-dimensional network structure formed by hydrophilic or amphiphilic polymer chains through physical or chemical crosslinking (Maddiboyina et al., 2022). It is an aqueous dispersion with good swelling properties and biocompatibility. And it is easy to synthesize and functionalize, is controllable in size, and has efficient drug loading capacity and good stability (Li et al., 2021b). According to the favorable characteristics, NG can be utilized as an ideal medical nanocarrier, which can integrate targeted molecules (Liang et al., 2016; Yang et al., 2019), therapeutic drugs (Peng et al., 2020; Li et al., 2021; Wang et al., 2021) and imaging reagents (Theune et al., 2019; Zhou et al., 2020) into one nanosystem.

Due to the excellent drug loading capacity of NGs, the SPECT imaging and radiotherapeutic property of radionuclide ^131^I, this study intends to build a NG functional platform based on polyethyleneimine (PEI) to realize accurate tumor diagnosis and high-efficient treatment. The synthesis steps were as follows. PEI and bisacrylamide (BIS) were reacted *via* Michael-Addition reaction to produce PEI-based NGs (P.NH_2_ NGs). Then 3-(4’-hydroxyphenyl) propionic acid N-hydroxysuccinimide (HPAO) was covalently cross-linked as a radionuclide linking molecule. Further modification of PEGylated phenylboronic acid (PBA) endowed the P.NH_2_ NGs with specific targeting to sialylated epitopes overexpressed on the surface of diversities of tumor cells. Finally, the remaining amino groups on the surface of PEI were acetylated and labeled with radionuclide ^131^I to obtain the final functionalized NGs. The resulting ^131^I-PBA-PHP NGs can specifically target breast cancer cells and efficiently aggregate into xenograft breast adenocarcinoma for tumor SPECT imaging and specific radiotherapy. This study provides an alternative strategy for clinical integrated diagnosis and treatment of breast cancer.

## Methods

### Evaluation of cellular uptake efficiency

In order to detect the intracellular intake of different NGs by cells, fluorescein isothiocyanate (FI) was modified on the surface of the PHP NGs and PBA-PHP NGs. The endowed fluorescence characteristics of PHP NGs and PBA-PHP NGs can be detected by flow cytometry. A density of 2 × 10^5^ cells per well were seeded into a 12-well plate and cultured for a period of time to guarantee 80% cell adherence. The culture medium was replaced by 1 ml DMEM containing 100 μL PHP NGs and PBA-PHP NGs. The final concentration of each NGs was 50 and 100 μg/ml, respectively. Incubation for another 6 h, all the NGs were sucked out and cleaned with PBS for 3 times. After trypsin suspension and mild centrifugation, the cells were dissolved in 1 ml PBS and analyzed by flow cytometry (BD Biosciences, Franklin lake, NJ, United States).

Additionally, the radiotherapy effect of radioactive ^131^I-PHP and ^131^I-PBA-PHP NGs was also evaluated. The subsequent operation steps were the same as described in Cytotoxicity Assay section (Supplementary Material), except that the additive materials were replaced with radioactive NGs. Similarly, the absorption value of the orifice plate at 450 nm was measured.

### Single-photon emission computed tomography imaging of tumor *in vivo*

Female BALB/C nude mice (about 20 g, 3–4 weeks) were subcutaneously inoculated with 4T1 cells into the right shoulder of a mouse with a density of 2×10^6^ cells/mouse. When the tumor grew to the size of 0.3–0.5 cm^3^, the mice were kept for further use. Notably, all the tumor bearing mice were fed drinking water containing 1% potassium iodide for one week prior to SPECT imaging to reduce thyroid uptake of ^131^I-labeled NGs. Before SPECT imaging, tumor bearing mice were anesthetized with sodium pentobarbital (40 mg/kg) through intraperitoneal injection. Then, the prepared ^131^I-PHP NGs and ^131^I-PBA-PHP NGs were injected into mice *via* a tail vein. At 30 min, 1, 2, 4, 6, 8, 12, and 16 h post-injection, SPECT imaging of tumor bearing mice *in vivo* were recorded. At 16 h post-injection, the tumor-bearing mice were randomly selected from each group and died of excessive anesthesia. The main organs and tumor tissues were collected to test their radioactive activities.

### Study on tumor radiotherapy *in vivo*

Similarly, tumor models were established in accordance with the above methods. When the tumors grew to the favorable size, normal saline (NS) solutions containing ^131^I-PHP NGs (1 mg/ml in NS, 200 μL) and ^131^I-PBA-PHP NGs (1 mg/ml in NS, 200 μL), respectively, were injected into the mice through a tail vein every 3 days (a total of four injections). NS (200 μL), no radioactive PHP NGs (1 mg/ml in NS, 200 μL) and PBA-PHP NGs (1 mg/ml in NS, 200 μL) were set as control. After that, tumor growth volume and body weight were monitored every 3 days. The tumor sizes were measured as follows: V = 0.5 × x × y^2^ (x represent the longest axis and y represent the shortest axis of the tumor). The relative tumor volume was calculated as below: ΔV% = V/V_0_ ×100%, where V_0_ is the volume before injection and V is the tumor volume at different time points. After the treatment, the survival period of the tumor bearing mice was continued to be monitored, and the death of each group was recorded for survival statistics.

### Pathologic analysis

The tumor bearing mice with different treatments (NS, PHP, PBA-PHP, ^131^I-PHP, and ^131^I-PBA-PHP NGs) were sacrificed according to the guidelines for the euthanasia of animals at 15 days and the tumorous tissues were collected. Hematoxylin & eosin (H&E) and terminal deoxynucleotidyl transferase-mediated dUTP-biotin nick end labeling (TUNEL) staining were performed. Finally, the apoptotic cells from TUNEL staining samples were observed and photographed by a Leica DM IL LED inverted phase contrast microscope, and the apoptotic cells were counted by free selection in the picture.

### Long-term biotoxicity

The long-term biotoxicity of the tumor model was evaluated after 15 days of materials injection. All mice were anesthetized. Major organs including lung, liver, spleen, kidney, and heart were harvested, fixed, embedded with paraffin, and followed by the H&E staining. Finally, the staining sections of tissues and organs were observed by a phase contrast microscope.

### Statistical analysis

The significant difference of the experimental data was analyzed through one-way ANOVA method, *p < 0.05, **p < 0.01, and ***p < 0.001, respectively.

## Results and discussion

### Characterization of ^131^I-PBA-PHP NGs

**SCHEME 1:**
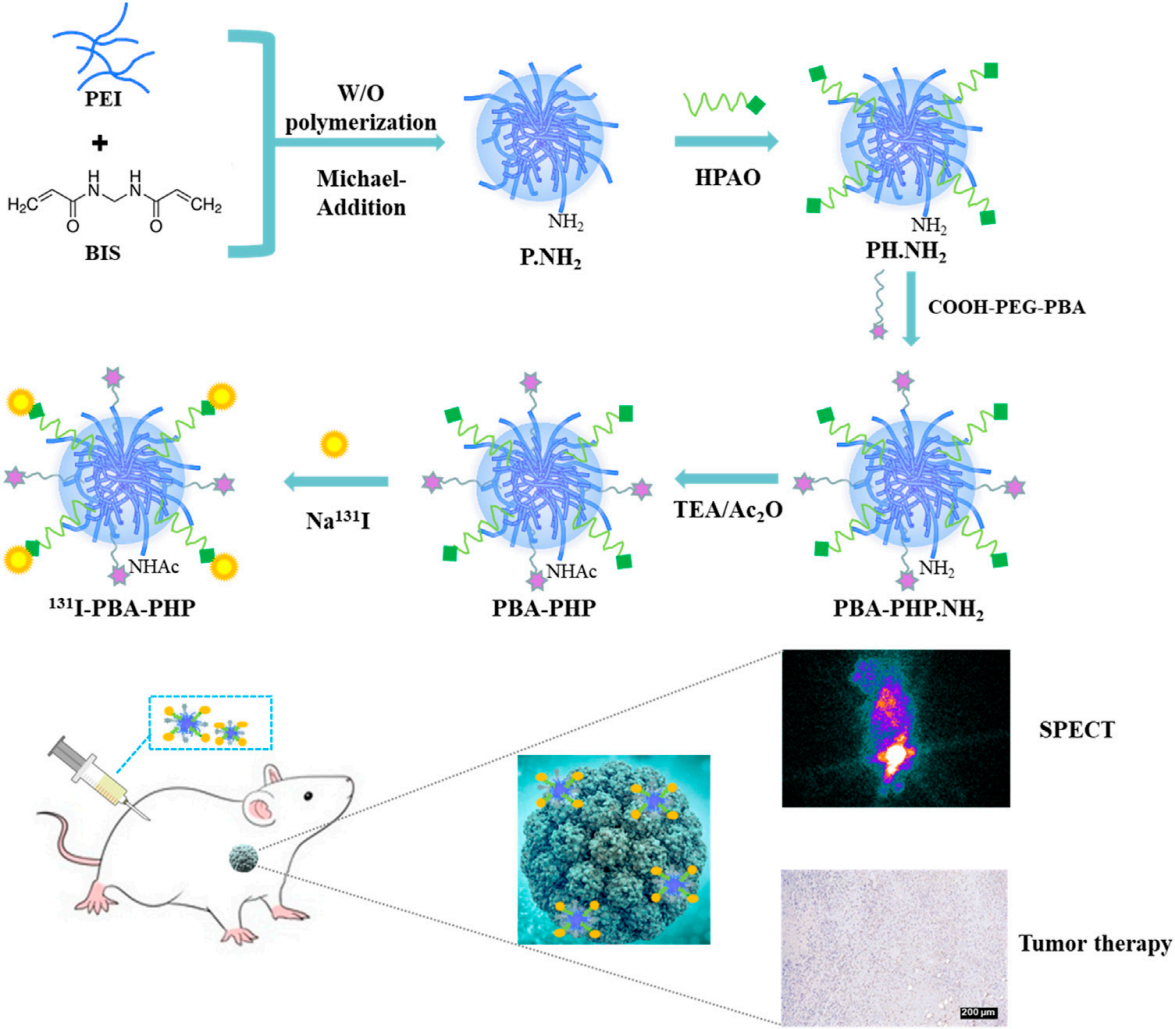
Illustration of ^131^I-PBA-PHP NGs preparation for SPECT imaging and radiotherapy of xenograft breast adenocarcinoma.

Through the water/oil (W/O) polymerization and Michael-Addition reactions, the initial P.NH_2_ NGs was synthesized by cross-linking PEI with BIS. Subsequently, the generated P.NH_2_ NGs reacted with HPAO and as-prepared COOH-PEG-PBA by means of N-hydroxysuccinimide ester group of HPAO and carboxyl group of COOH-PEG-PBA to form PBA-PHP.NH_2_ NGs. Followed by acetylation of the remaining amino groups of PBA-PHP.NH_2_ NGs and labeling ^131^I onto the PBA-PHP.NH_2_ NGs *via* HPAO, the final ^131^I-PBA-PHP NGs were constructed (Scheme 1).

**FIGURE 1:**
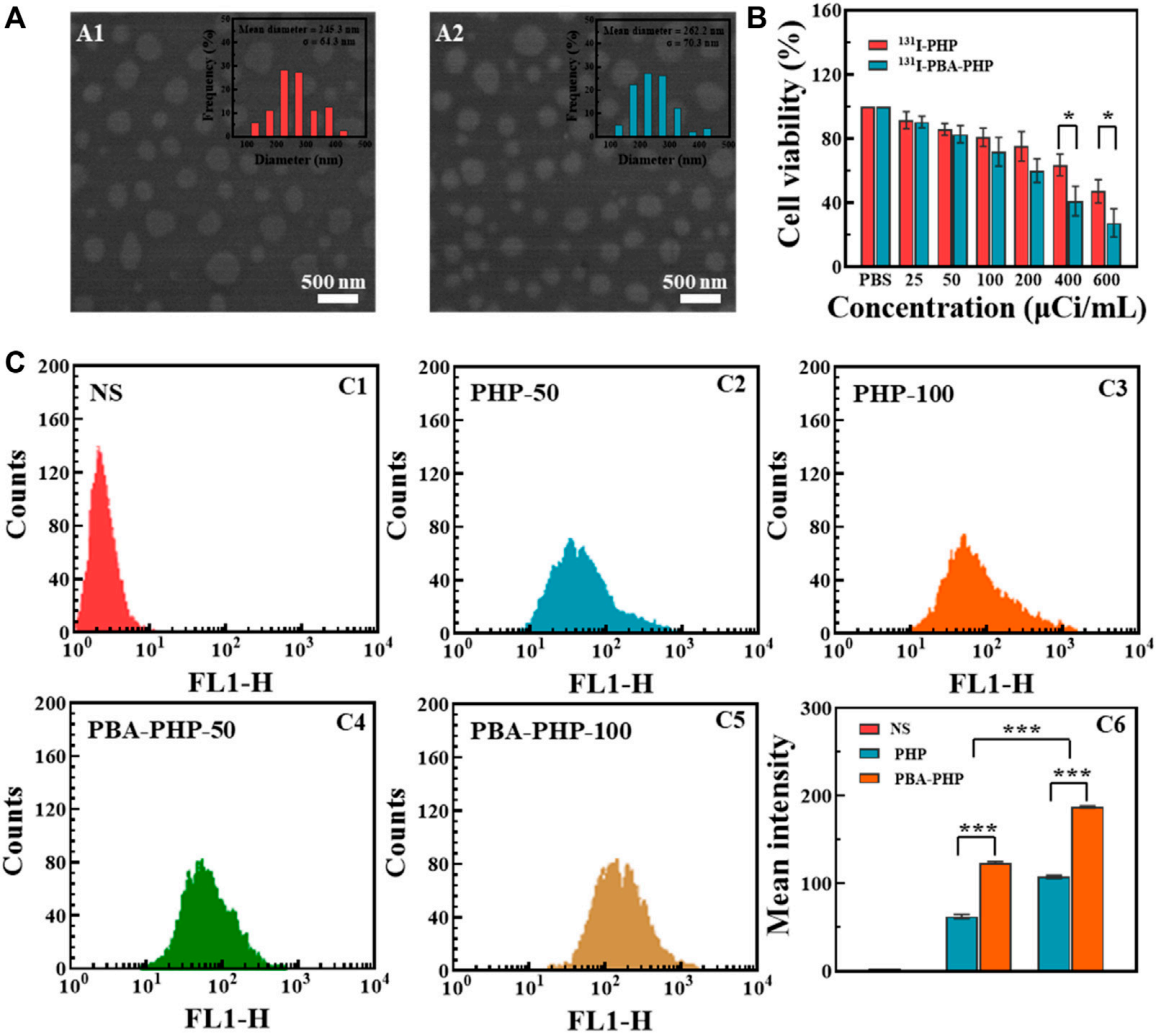
**(A)** SEM images of (A1) PHP NGs and (A2) PBA-PHP NGs. **(B)** CCK-8 assay of 4T1 cells incubated with ^131^I-PHP NGs and ^131^I-PBA-PHP NGs at different radioactive concentrations for 24 h, respectively. **(C)** Flow cytometry tests of 4T1 cells treated with C1: NS; C2: PHP NGs (50 μg/ml); C3: PHP NGs (100 μg/ml); C4: PBA-PHP NGs (50 μg/ml)and C5: PBA-PHP NGs (100 μg/ml) for 6 h, respectively. C6: The statistical analysis of the mean fluorescence of cells treated with PHP NGs and PBA-PHP NGs. *p < 0.05, ***p < 0.001.

A series of characterization methods were carried out to analyze the composition, morphology, size and basic properties of ^131^I-PBA-PHP NGs. The intermediate products COOH-PEG-PBA and PH.NH_2_ NGs were characterized by ^1^H NMR first (Supplementary Figure S1). The distinct peaks at 7.7–7.8 ppm and 3.3–3.7 ppm in the ^1^H NMR spectrum of PBA-PEG-COOH were associated with the typical characteristic protons on the phenyl group of PBA and the alkoxy of PEG respectively, reflecting the successful formation of COOH-PEG-PBA (Supplementary Figure S1A). Through integration the characteristic peaks of PBA and PEG, it can be figured out every PEG conjugated with 0.4 PBA. And the district peaks at 6.8–7.1 ppm and 2.0–3.2 ppm in the ^1^H NMR spectrum of PH.NH_2_ NGs corresponding to the characteristic protons on the phenol group of HPAO and alkyl skeleton of PEI, revealing the successful conjugation of HPAO onto PEI (Supplementary Figure S1B). Through integration the characteristic peaks of HPAO and PEI, it can be figured out every PEI conjugated with 9.6 HPAO. As a kind of cationic polymer, P.NH_2_ NGs tended to bind protons *via* surface amino group. Hence, P.NH_2_ NGs demonstrated the relatively high Zeta potential (60.0 ± 1.2 mV). After modification with HPAO, COOH-PEG-PBA or *m*PEG-COOH, and acetic anhydride, the generated series of products (PH.NH_2_ NGs, PHP.NH_2_ NGs, PBA-PHP.NH_2_ NGs, PHP NGs, and PBA-PHP NGs) have different Zeta potentials. Due to the gradually reducing of remaining amino group on the surface of P.NH_2_ NGs, different NGs displayed the decreasing trend of Zeta potential (Supplementary Figure S2A). And the hydrodynamic diameters of PHP NGs and PBA-PHP NGs were determined to be 342.6 ± 11.5 nm and 389.2 ± 7.1 nm, respectively, which were within acceptable size range (Supplementary Figure S2B). The morphologies and sizes of the formed PHP NGs and PBA-PHP NGs were recorded by SEM. The mean diameters of PHP NGs and PBA-PHP NGs with the spheroidal structure could be analyzed to be 245.3 ± 64.3 nm and 262.2 ± 70.3 nm, respectively (Figure 1A). It was remarkable that the sizes of PHP NGs and PBA-PHP NGs measured by DLS were larger than that detected by SEM, which was attributed to the basic swelling property of NGs in water within the DLS testing process, rather than the compression of NG networks during the drying process for the SEM recording. After labeling ^131^I onto PHP NGs and PBA-PHP NGs, the radiochemical yields of ^131^I-PHP NGs and ^131^I-PBA-PHP NGs were reached to 71.5% ± 2.8% and 74.4% ± 5.9%, respectively (Supplementary Figure S3A). After purification with ITLC, the radiochemical purities of ^131^I-PHP NGs and ^131^I-PBA-PHP NGs were approached to 99%. Followed by radiostability characterization at various time points, both of ^131^I-PHP NGs and ^131^I-PBA-PHP NGs displayed the satisfactory radiostability, which have the relatively high radiochemical purities (> 90%) even after labeling for 24 h (Supplementary Figure S3B).

### *In vitro* cancer cell inhibition efficacy

After incubation with PHP NGs, PBA-PHP NGs, ^131^I-PHP NGs, and ^131^I-PBA-PHP NGs for 24 h, CCK-8 assay was carried out to evaluate their inhibition efficiency towards 4T1 cells. PHP NGs and PBA-PHP NGs treated cells had high cell viability (>95%) even when the concentration went up to 200 μg/ml, indicating the favorable biocompatibility of PHP NGs and PBA-PHP NGs (Supplementary Figure S4). However, ^131^I-PHP NGs and ^131^I-PBA-PHP NGs presented a certain degree of inhibition towards 4T1 cells, which was solely attributed to the radiotherapeutic effect of ^131^I. And it revealed the radioactive dose-dependent cancer cell inhibition, the higher radioactive concentration, the lower cell viability the materials caused. And the radiotherapeutic efficiency of ^131^I-PHP NGs and ^131^I-PBA-PHP NGs towards 4T1 cells are different. ^131^I-PBA-PHP NGs showed significantly higher inhibition efficiency than ^131^I-PHP NGs towards 4T1 cells, especially at radioactive concentration of 400 μCi/ml and 600 μCi/ml (*p* < 0.05). The good inhibition effect of ^131^I-PBA-PHP NGs towards 4T1 cells probably attribute to the PBA-mediate targeting ability, which could specifically bind to sialylated epitopes overexpressed on the surface of 4T1 cells (Figure 1B).

### Evaluation of cellular uptake efficiency

To assess the PBA mediated targeting ability, FI was utilized as a fluorescent tracer to modify onto the PBA-PHP NGs after HPAO modification. As control, the FI modified PHP NGs were also synthesized following the same steps, subsequently. Through the flow cytometry analysis, the mean fluorescence intensity of 4T1 cells was enhanced with the increasing concentration of PHP NGs and PBA-PHP NGs, suggesting the concentration-dependent cellular uptake manner. And the PBA-PHP NGs treated cells exhibited much higher fluorescence intensity than the PHP NGs treated cells at the same concentration (*p* < 0.001, Figure 1C). The high fluorescence intensity of 4T1 cells after treated with PBA-PHP NGs reveals the targeting efficiency of PBA.

### Single-photon emission computed tomography imaging of tumor *in vivo*

**FIGURE 2:**
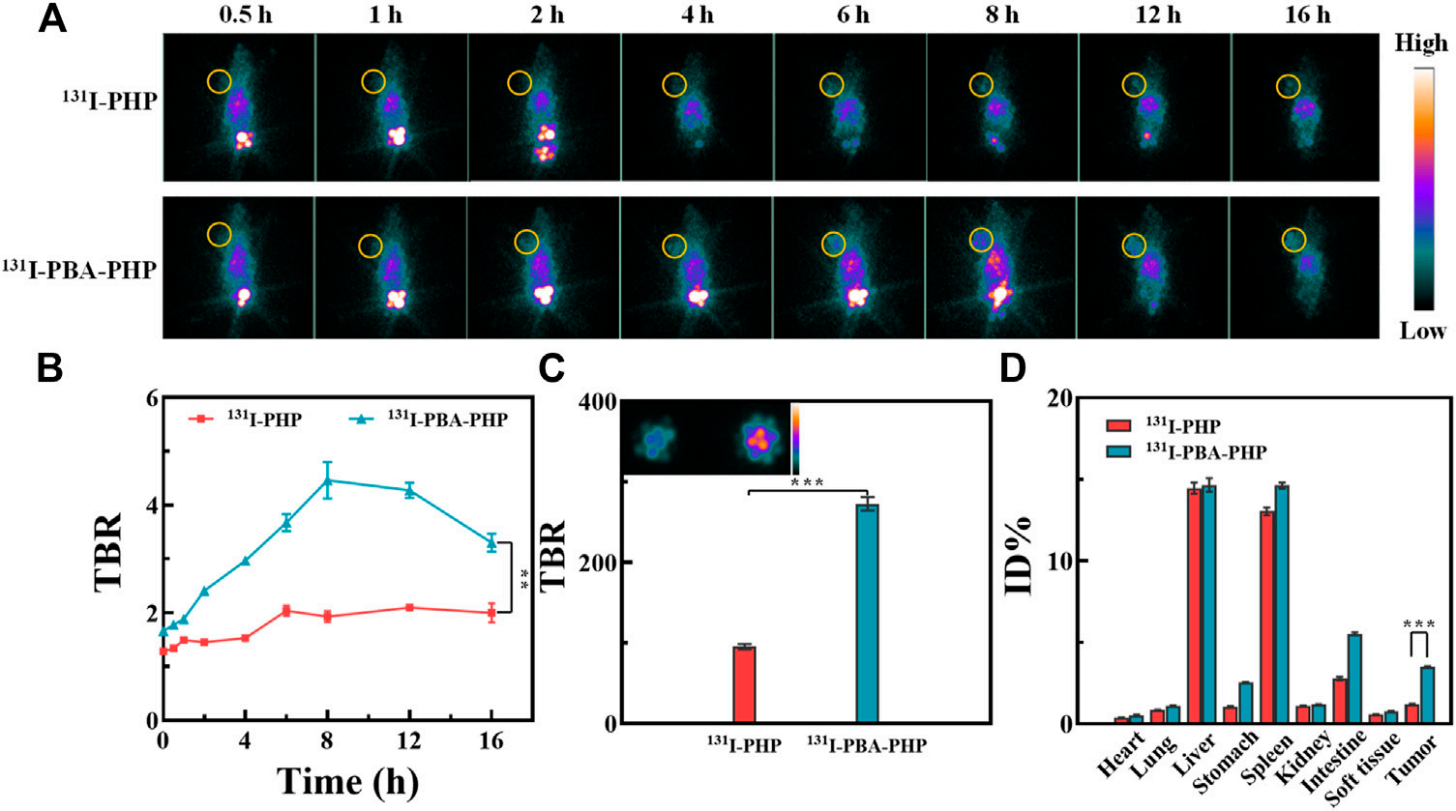
**(A)** SPECT images and **(B)** the corresponding TBR of 4T1 tumor bearing mice post-injection of ^131^I-PHP NGs and ^131^I-PBA-PHP NGs at various time points, respectively. **(C)** The SPECT images of *ex vivo* tumors at 16 h post-injection of different materials. **(D)** The relative SPECT signal intensities of major organs at 16 h post-injection of different materials. ^#^The yellow circle points out the tumor site. **p < 0.01, ***p < 0.001.

Based on the inherent property of ^131^I, the *in vivo* SPECT imaging of ^131^I-PBA-PHP NGs treated tumor bearing mice should be further investigated. Among the SPECT images of tumor bearing mice with different administrations, the prominent tumor signals could be found in the ^131^I-PBA-PHP NGs treated tumor bearing mice from 2 h to 16 h post-injection. In comparison, the ^131^I-PHP NGs treated tumor bearing mice had the relatively weak tumor SPECT signals from 2 h to 16 h post-injection (Figure 2A). Due to the high imaging sensitivity of radionuclide ^131^I, the differences of the imaging signals between ^131^I-PBA-PHP NGs and ^131^I-PHP NGs treated tumor bearing mice could be highlighted, suggesting that the larger amount of ^131^I-PBA-PHP NGs could be delivered into tumor site than ^131^I-PHP NGs based on the PBA-mediated targeting effect. Thereafter, the tumor-to-background ratio (TBR) at different time point was also recorded through collecting the SPECT signal intensities of corresponding sites. The quantitative analysis result manifested that the TBR of ^131^I-PBA-PHP NGs treated tumor bearing mice had the sharply raised stage from 0.5 h to 8 h and the gradually declined stage from 8 h to 16 h. The peak value of TBR could be found at 8 h post-injection of ^131^I-PBA-PHP NGs. However, the ^131^I-PHP NGs treated tumor bearing mice had the almost constant TBR within 16 h post-injection. The TBR of ^131^I-PBA-PHP NGs treated tumor bearing mice was significantly higher than that of ^131^I-PHP NGs treated tumor bearing mice at the same time point within 16 h (*p* < 0.01, Figure 2B). The quantitative analysis result is consistent with the SPECT images of tumor bearing mice, further suggesting the targeting effect of PBA moiety. The SPECT imaging of *ex vivo* tumors further verified the different tumor accumulation of different materials. The inserted SPECT image of *ex vivo* tumors clearly revealed the signal differences between ^131^I-PBA-PHP NGs and ^131^I-PHP NGs treated tumor bearing mice. And the *ex vivo* tumor signal intensity of ^131^I-PBA-PHP NGs treated tumor bearing mice is almost 2.9 folds that of ^131^I-PHP NGs treated tumor bearing mice (Figure 2C). Finally, the SPECT signal intensities of major organs from the treated tumor bearing mice were collected. The results displayed the biodistribution of different materials *in vivo*. Large amounts of ^131^I-PBA-PHP NGs and ^131^I-PHP NGs could accumulate into liver, spleen, and intestine. A small quantity of ^131^I-PBA-PHP NGs and ^131^I-PHP NGs could accumulate into heart, lung, stomach, kidney, soft tissue, and tumor. Moreover, ^131^I-PBA-PHP NGs had the significantly higher tumor accumulation than that of ^131^I-PHP NGs (*p* < 0.001), reflecting the PBA induced high tumor accumulation of ^131^I-PBA-PHP NGs (Figure 2D).

### *In vivo* tumor inhibition efficacy

**FIGURE 3:**
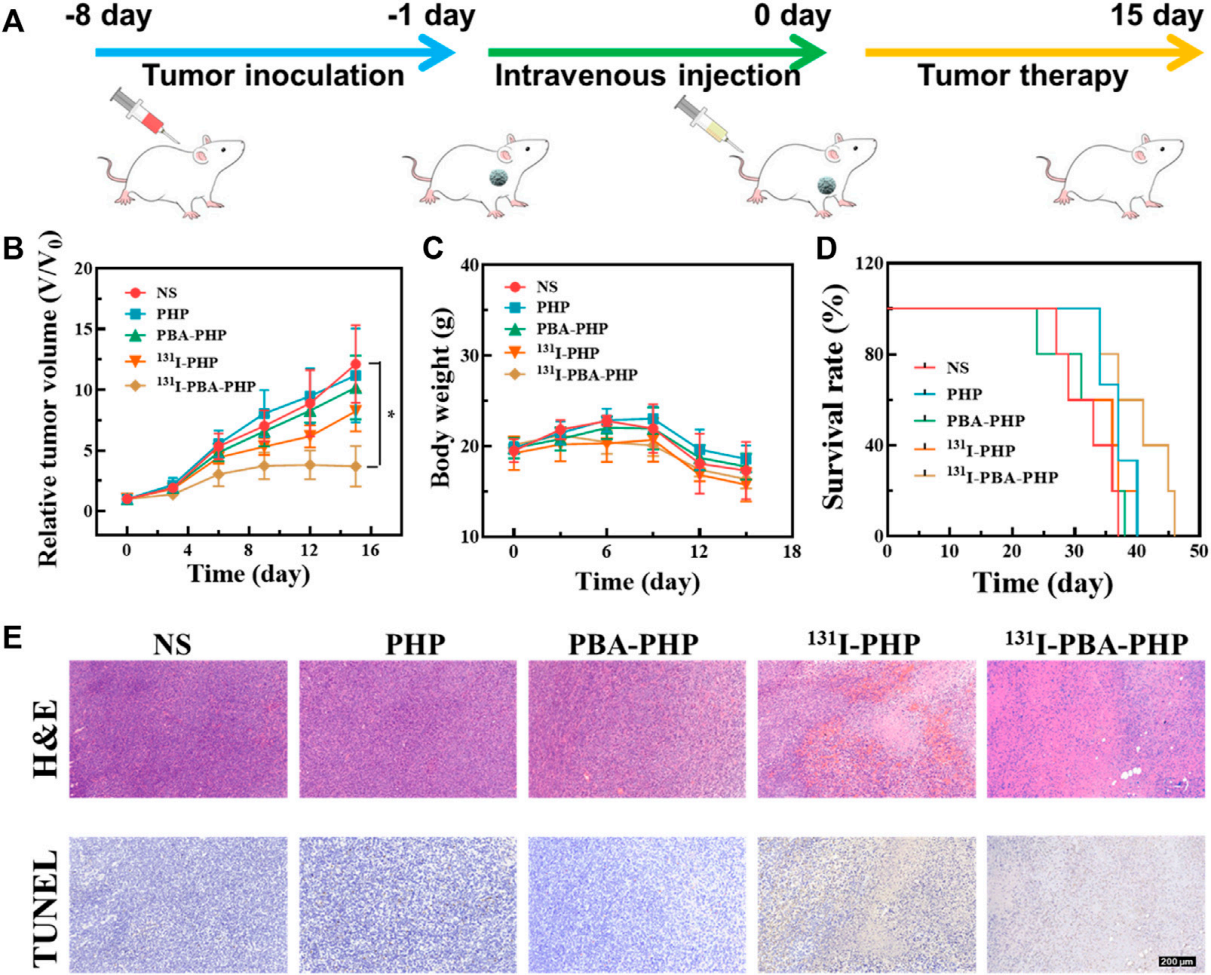
**(A)** Illustration of treatment process of tumor inoculation, intravenous injection, and tumor therapy of 4T1 tumor bearing mice. **(B)** The relative tumor volume, **(C)** body weight, **(D)** survival rate, and **(E)** representative tumor H&E and TUNEL images of mice with various treatments. The scale bar inserted in panel represents 200 μm. *p < 0.05.

The tumor inhibition efficacy of ^131^I-PBA-PHP NGs was further studied from the perspectives of tumor growth, body weight change, and survival rate statistic. The tumor bearing mice treated with NS, PHP NGs, PBA-PHP NGs, and ^131^I-PHP NGs were set as control groups. Through tumor inoculation, materials injection, and a period of therapy, the tumor inhibition efficacy was assessed (Figure 3A). In terms of tumor growth, the NS, PBA-PHP NGs, and PHP NGs treated tumor bearing mice had the similar tumor growth trend and the tumors grew fast. Due to the radiotherapeutic function of ^131^I, ^131^I-PHP NGs group possessed a certain anti-tumor efficacy, and its tumor growth trend was relatively slower than that of the mice with injection of NS, PBA-PHP NGs, and PHP NGs. Above all, the tumor bearing mice with injection of ^131^I-PBA-PHP NGs displayed the slowest tumor growth trend among all the groups. Through statistical analysis, the tumor volume of ^131^I-PBA-PHP NGs group was significantly smaller than that of NS group after 15 days’ therapy (*p* < 0.05). The best anti-tumor efficacy of ^131^I-PBA-PHP NGs among all the materials manifested the role of PBA, which could cause the high tumor accumulation of ^131^I-PBA-PHP NGs *via* the PBA-mediated targeting effect (Figure 3B). As for body weight changes of tumor bearing mice with injection of NS, PHP NGs, PBA-PHP NGs, ^131^I-PHP NGs, and ^131^I-PBA-PHP NGs, all the mice had the similar body weight change trend without enormous weight fluctuation, revealing their great biocompatibility (Figure 3C). The survival rate of mice reflected the anti-tumor efficiency of ^131^I-PBA-PHP NGs indirectly. ^131^I-PBA-PHP NGs group obviously prolonged the lifetime of mice. Until 47th day, all the mice injected with ^131^I-PBA-PHP NGs were dead. However, the mice with treatment of NS, PHP NGs, PBA-PHP NGs, and ^131^I-PHP NGs respectively were dead before 40th day. The superior therapeutic efficacy suggested the PBA-mediated targeted delivery of ^131^I-PBA-PHP NGs towards tumor (Figure 3D).

### Pathologic analysis

After the whole treatment, the pathological analysis was performed to deeply assess the anti-tumor efficiency of ^131^I-PBA-PHP NGs *via* H&E staining and TUNEL assay of tumor. The tumor necrosis and apoptosis could be stained pink and brown respectively in H&E and TUNEL images of tumor, From the H&E and TUNEL images of tumor, it was evident that the ^131^I-PBA-PHP NGs group presented largest region of tumor necrosis and apoptosis among all the five groups (Figure 3E). Subsequently, through quantitatively analyzing the tumor apoptosis rate from the TUNEL staining images (Supplementary Figure S5), it could be ranked as following order PHP NGs (9.2%) < PBA-PHP NGs (18.8%) < ^131^I-PHP NGs (59.6%) < ^131^I-PBA-PHP NGs (76.6%), clearly showing that the area of tumor necrosis and apoptosis of ^131^I-PBA-PHP NGs group was larger than that of ^131^I-PHP NGs group (*p* < 0.01). Through the above comprehensive investigation, ^131^I-PBA-PHP NGs possess good anti-tumor efficiency, which is attributed to the targeting effect of PBA and radiotherapeutic function of radioactive ^131^I. In the meantime, the major organs of the treated tumor bearing mice were harvested and H&E staining was performed. Through observing the H&E images of major organs, there was no visible necrosis or abnormality area, manifesting the favorable biosafety of ^131^I-PBA-PHP NGs *in vivo* (Supplementary Figure S6).

## Conclusion

On the whole, we constructed and synthesized the multifunctional ^131^I-PBA-PHP NGs for SPECT imaging and radiotherapy of xenograft breast adenocarcinoma. Through the W/O polymerization and Michael-Addition reactions, P.NH_2_ NGs were prepared. Followed by reacting with HPAO, COOH-PEG-PBA, acetylation and ^131^I labeling, the ^131^I-PBA-PHP NGs were constructed. The generated ^131^I-PBA-PHP NGs with a spherical structure and favorable radiostability own targeting property towards sialylated epitopes overexpressed on the surface of 4T1 cells. With linking of PBA, the PBA-PHP NGs manifest the improved cellular uptake in comparison with PHP NGs. Above all, the formed ^131^I-PBA-PHP NGs enable the targeted SPECT imaging and radiotherapy of 4T1 tumor bearing mice *in vivo*. The excellent targeting moiety PBA has the great potential to be conjugated onto the other polymers to build intelligent nanosystems for tumor theranostic.

## Data Availability

The original contributions presented in the study are included in the article/Supplementary Material, further inquiries can be directed to the corresponding authors.
