# Supplementary material for: Phenylboronic acid conjugated multifunctional nanogels with 131I-labeling for targeted SPECT imaging and radiotherapy of breast adenocarcinoma
Source: Front Bioeng Biotechnol. 2022 Jul 22;10:973141. doi: 10.3389/fbioe.2022.973141 (PMC9359430; doi:10.3389/fbioe.2022.973141)
Supplement: Supplementary file 1 [file DataSheet1.docx]

Supplementary Material

# Materials and Methods

## Materials

Polyethyleneimine (PEI, Mw = 25000), 1-(3-(dimethylamino)propyl)-3-ethylcarbodiimide hydrochloride (EDC), N-hydroxysuccinimide (NHS), triethylamine (TEA) and acetic anhydride (Ac_2_O) were purchased from Sigma-Aldrich (St. Louis, MO). Bisacrylamide (BIS), chloramine-T trihydrate, sodium pyrosulfite (Na_2_S_2_O_5_), 3-(4’-hydroxyphenyl) propionic acid N-hydroxysuccinimide (HPAO), and 4-carboxyphenylboronic acid (PBA) were obtained from J&K Scientific (Beijing, China). COOH-PEG-NH_2_ and *m*PEG-COOH were from Shanghai Yanyi Biological Technology Co., LTD (Shanghai, China). Na^131^I reagent was purchased from Shanghai Xinke Pharmaceutical Co., LTD (Shanghai, China). Dulbecco’s modified eagle medium (DMEM), Fetal bovine serum (FBS), penicillin, and streptomycin were acquired from Hangzhou Jinuo Biomedical Technology Co., Ltd. (Hangzhou, China). Cell Counting Kit 8 (CCK-8) was supplied by Dojindo Molecular Technologies, Inc. (Shanghai, China). Mouse mammary tumor cells (4T1 cells) were obtained from the Institute of Biochemistry and Cell Biology, the Chinese Academy of Sciences (Shanghai, China).

## Synthesis of P. NH_2_ NGs

P.NH_2_ NGs were synthesized by an inverse emulsion method. BIS was used as the cross-linker for Michael addition reaction. Briefly, the water solution (2.5 mL) containing PEI (136 mg) and BIS (16 mg) were added into the toluene solution containing Span 80 (600 mg, 30 mL) under stirring for 30 min. Afterwards, the mixture was emulsified by a homogenizer (XL2000, Division of QSonica, Newtown, CT) for 30 min with an output wattage of 20 W. Then, 1.2 mL of TEA was dropwise added into the above emulsion to initiate the cross-linking reaction between BIS and PEI under stirring. After that, the emulsion was kept stirring for 24 h, centrifuged at 10,000 rpm for 15 min, redispersed with methanol, and dialyzed against water. The final obtained P.NH_2_ NGs were kept at 4 ^o^C for further use.

## Synthesis of ^131^I-PBA-PHP NGs

COOH-PEG-NH_2_ was first reacted with the activated PBA to obtain COOH-PEG-PBA. The above P.NH_2_ NGs aqueous solution (20 mL, 10 mg/mL) was mixed with DMSO solution containing HPAO (10 mL, 2.53 mg/mL) under stirring for 24 h to synthesize PEI-HPAO NGs (PH.NH_2_ NGs). Then, the PH.NH_2_ NGs were reacted with the activated COOH-PEG-PBA or *m*PEG-COOH with a molar ratio of 1:10, and followed by the acetylation. The corresponding product was abbreviated as PBA-PHP NGs or PHP NGs. Finally, ^131^I labelled PBA-PHP NGs (^131^I-PBA-PHP NGs) and ^131^I labelled PHP NGs (^131^I-PHP NGs) were prepared by radioactive ^131^I labeling according our previous report(Zhu et al. 2015).

## Characterization

The prepared PBA-PEG-COOH and PH.NH_2_ NGs were characterized by ^1^H NMR to ensure the modification of PBA and HPAO. ^1^H NMR spectroscopy was carried out using a Bruker AV400 NMR spectrometer. All materials (~ 3 mg) were dissolved in D_2_O before analysis. Field emission scanning electron microscopy (FESEM) was observed using a Phenom ProX scanning electron microscope (Thermo fisher scientific, MA) at a working voltage of 15 kV. Samples were prepared by depositing the as-prepared NPs aqueous dispersion onto a silicon slice. Dynamic light scattering (DLS) and Zeta potential measurements were tested by using a Zetasizer Nano-ZS Nanoseries (Malvern Instruments, Worcestershire, UK) equipped with a standard 633 nm HeNe laser. After ^131^I labeling, the radiochemical purities of ^131^I-PHP NGs and ^131^I-PBA-PHP NGs dissolved in PBS at room temperature at different time points (such as 1, 3, 6, 15, and 24 h) were measured by instant thin-layer chromatography (ITLC), respectively to determine their radiostability.

## Cytotoxicity Assay

The cytotoxicity of the synthesized PHP NGs, PBA-PHP NGs, ^131^I-PHP NGs, and ^131^I-PBA-PHP NGs was evaluated by the CCK-8 viability assay. 4T1 cells at a density of 8×10^3^ per well in 96-well plate were pre-cultured in DMEM, with 10% FBS, 100 U/mL penicillin, and 100 U/mL streptomycin. Cells was cultured in an incubator containing 95% air and 5% CO_2_ at 37 °C overnight before testing. The medium was discarded and replaced with flesh medium containing various amounts of PHP NGs and PHP-PBA NGs (0, 20, 40, 80, 120, 160, and 200 μg/mL) and various radioactive concentrated ^131^I-PHP NGs, and ^131^I-PBA-PHP NGs (0, 25, 50, 100, 200, 400, and 600 μCi/mL). After 24 h, the culture medium was replaced by 100 μL DMEM containing 10 μL CCK-8 reagent and co-incubated for another 30 min. The characteristic absorption value at a wavelength of 450 nm was tested using a PerkinElmer microplate reader (Boston, MA).

# Supplementary Figures


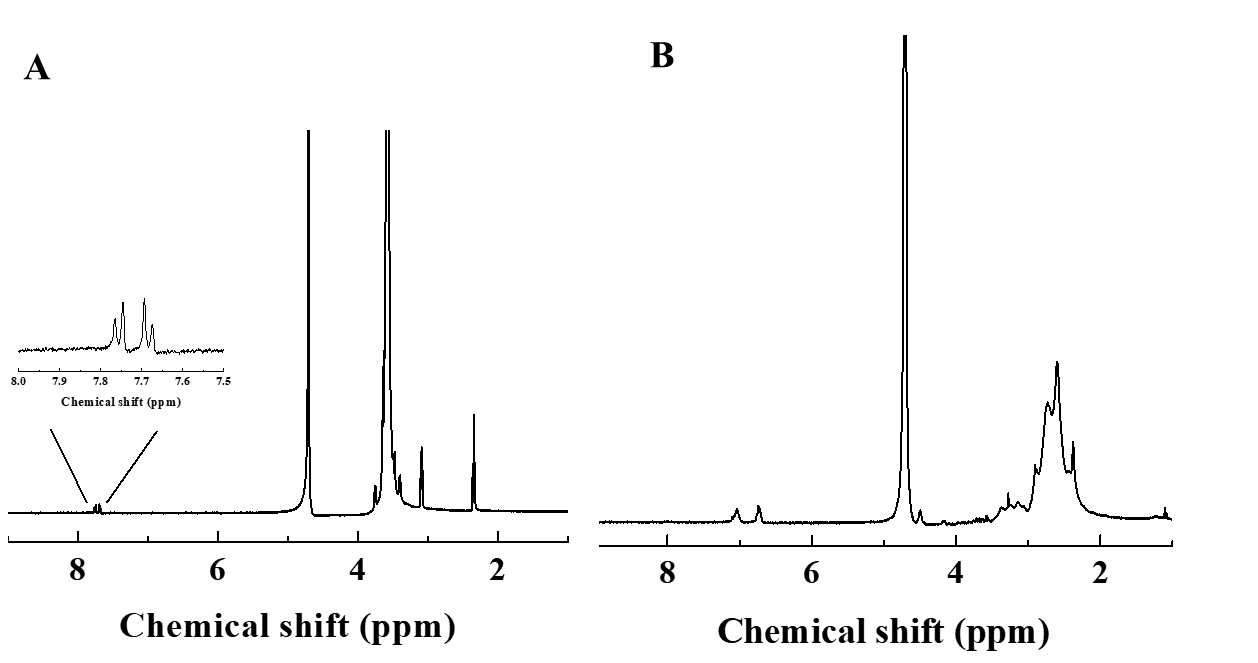


**FIGURE S1.** ^1^H NMR spectra of (A) COOH-PBA-PEG and (B) PH.NH_2_ NGs dispersed in D_2_O.

**FIGURE S2.** Zeta potentials (A) and hydrodynamic diameters (B) of different materials.


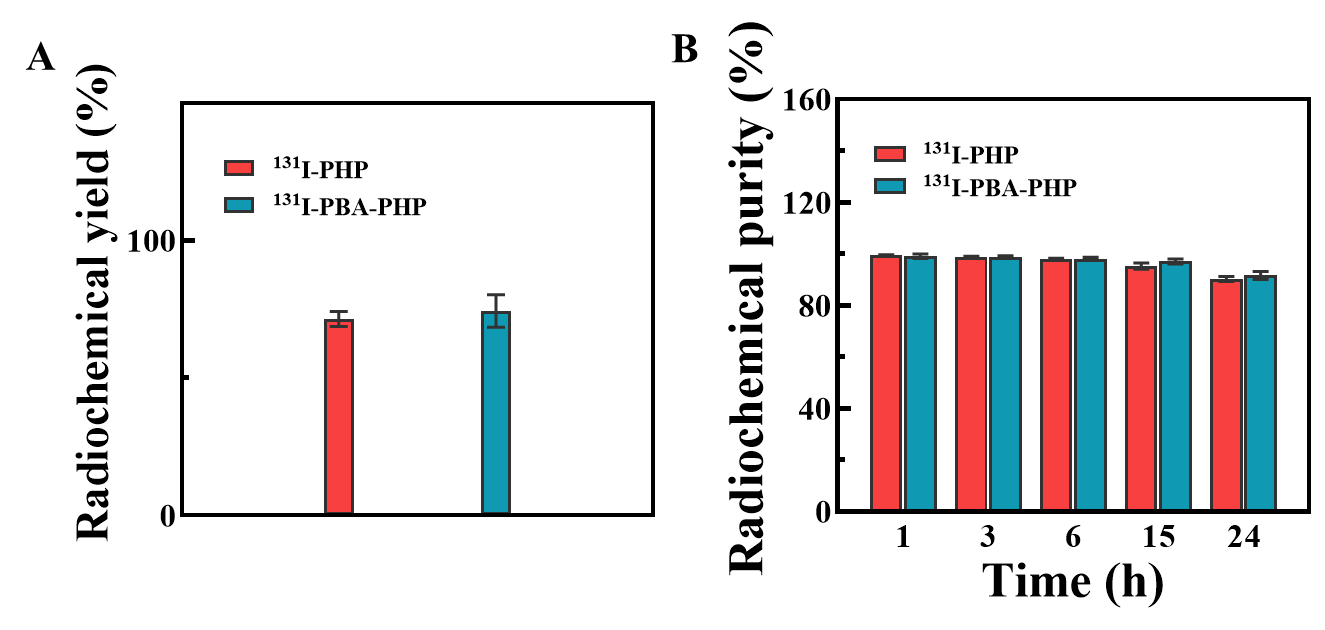


**FIGURE S3.** (A) Raidochemical yields of ^131^I-PHP NGs and ^131^I-PBA-PHP NGs; (B) radiochemical purities of ^131^I-PHP NGs and ^131^I-PBA-PHP NGs at different time points after ^131^I labeling.

**FIGURE S4.** CCK-8 assay of 4T1 cells incubated with PHP NGs and PBA-PHP NGs at different concentrations for 24 h, respectively.

**FIGURE S5.** Quantitative analysis of the apoptosis rate of tumor cells corresponding to TUNEL images. **p < 0.01.

**
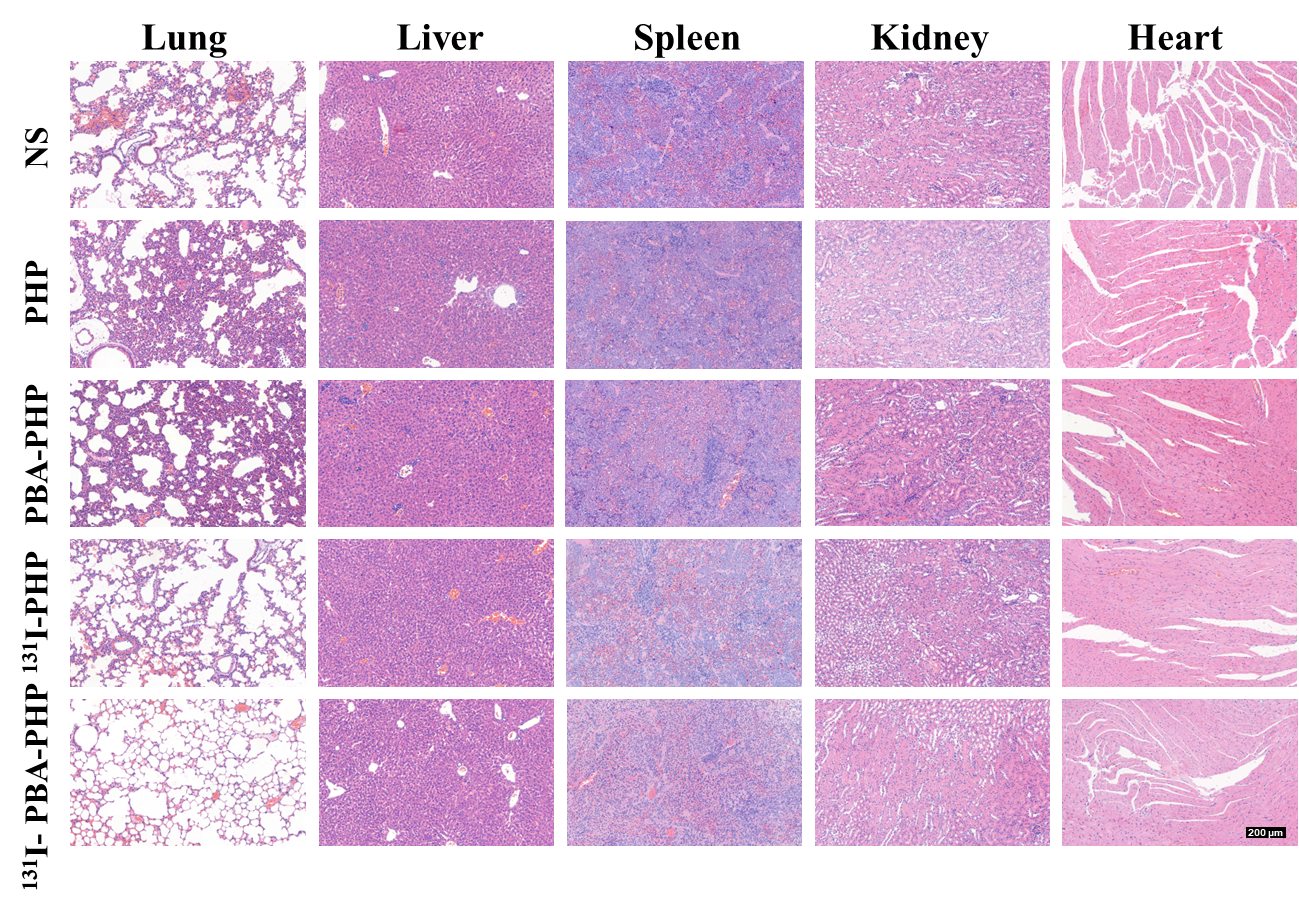
**

**FIGURE S6.** Representative H&E images of the major organs from the survived mice with various treatments. The scale bar inserted in the image represents 200 μm.

# Reference

Zhu, J., Zhao, L., Cheng, Y., Xiong, Z., Tang, Y., Shen, M., Zhao, J. & Shi, X. (2015). Radionuclide 131I-Labeled Multifunctional Dendrimers for Targeted Spect Imaging and Radiotherapy of Tumors. *Nanoscale* 7, 18169-18178. doi:10.1039/C5NR05585G
